# Fatigue after COVID‐19 in non‐hospitalized patients according to sex

**DOI:** 10.1002/brb3.2849

**Published:** 2023-01-09

**Authors:** Iwona Mazurkiewicz, Zaneta Chatys‐Bogacka, Joanna Slowik, Alicja Klich‐Raczka, Malgorzata Fedyk‐Lukasik, Agnieszka Slowik, Marcin Wnuk, Leszek Drabik

**Affiliations:** ^1^ Department of Neurology University Hospital in Krakow Krakow Poland; ^2^ Department of Neurology Jagiellonian University Medical College Krakow Poland; ^3^ Department of Periodontology, Preventive Dentistry and Oral Medicine, Institute of Dentistry, Faculty of Medicine Jagiellonian University Medical College Krakow Poland; ^4^ Department of Internal Medicine and Gerontology Jagiellonian University Medical College Krakow Poland; ^5^ Department of Internal Medicine and Geriatrics University Hospital in Krakow Krakow Poland; ^6^ Department of Pharmacology Jagiellonian University Medical College Krakow Poland; ^7^ Department of Cardiac and Vascular Diseases John Paul II Hospital Krakow Poland

**Keywords:** COVID‐19, fatigue, long covid, sex

## Abstract

**Background:**

Limited evidence exists on sex differences in post‐COVID fatigue among non‐hospitalized patients. Therefore, aim of the study was to evaluate the course of chronic fatigue symptoms in non‐hospitalized subjects with the SARS‐CoV‐2 infection, according to sex.

**Methods:**

Patients and staff from the University Hospital in Krakow anonymously and retrospectively completed neuropsychological questionnaire that included eight symptoms of chronic fatigue syndrome. The presence of these symptoms was assessed before COVID‐19 and 0–4, 4–12, and >12 weeks postinfection. The inclusion criteria were as follows: age 18 or more years, >12 weeks since the onset of the SARS‐CoV‐2 infection, and diagnosis confirmed by the RT‐PCR from anasopharyngeal swab.

**Results:**

We included 303 patients (79.53% women, 47.52% medical personnel) assessed retrospectively after a median of 30 (interquartile range: 23–35) weeks since the onset of symptoms. A higher prevalence of at least one chronic fatigue symptom was found in females in all time intervals after the onset of COVID‐19 compared to males (*p* < .036). Women, compared to men, more often experienced persistent fatigue, not caused by effort and persisting after rest (for <4 weeks, odds ratio [OR] = 2.31, 95% confidence interval [CI]: 1.13–4.73; for 4–12 weeks, OR = 1.95, 95% CI: 1.06–3.61), non‐restorative sleep (for <4 weeks, OR = 2.17, 95% CI: 1.23–3.81; for >12 weeks, OR = 1.95, 95% CI: 1.03–3.71), and sore throat (for <4 weeks, OR = 1.97, 95% CI: 1.03–3.78; for 4–12 weeks, OR = 2.76, 95% CI: 1.05–7.27). Sex differences in headache, arthralgia, and prolonged postexercise fatigue were observed only during the first 4 weeks (OR = 2.59, 95% CI: 1.45–4.60, OR = 2.97, 95% CI: 1.02–8.64, and OR = 1.87, 95% CI: 1.01–3.51, respectively). There were no differences between women and men in myalgia and self‐reported lymph node enlargement.

**Conclusions:**

The course of post‐COVID fatigue differs significantly between sexes in non‐hospitalized individuals with COVID‐19, with women more often suffering from persistent fatigue, not caused by effort and persisting after rest, non‐restorative sleep, and sore throat.

## Graphical abstract

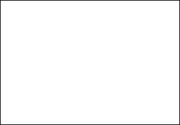

## 1 INTRODUCTION

Since its first identification among hospitalized patients with pneumonia in Wuhan, China (Zhu et al., 2020), the Coronavirus Disease 2019 (COVID‐19) has become an emerging medical problem around the world. Although a third of individuals affected with the Severe Acute Respiratory Syndrome coronavirus 2 (SARS‐CoV‐2) infection are asymptomatic (Bliddal et al., 2021), an increasing number of patients develop a wide spectrum of persistent symptoms, affecting multiple organs and systems, which has been collected under the term of “long COVID” and created the potential for a new pandemic (Fernández‐Lázaro et al., 2021). In particular, more than 60% of COVID‐19 survivors reported the presence of at least one symptom after 30 days since the onset of disease or hospitalization (Fernández‐de‐las‐Peñas et al., 2021), and up to 30% experienced deterioration in the quality of life related to the disease (Logue et al., 2021).

Previous studies have shown that fatigue constitutes the most important complication after COVID‐19 (Chen et al., 2022; Hellwig & Domschke, 2022), in both hospitalized and non‐hospitalized patients (Petersen et al., 2021). In a meta‐analysis of 19 studies, it was found that fatigue was reported by one third of individuals with the SARS‐CoV‐2 infection at any time point (Healey et al., 2022); however, according to some systematic reviews, its prevalence could be even higher, with more than 50% of affected patients (Lopez‐Leon et al., 2021). Postinfectious fatigue after COVID‐19 was shown to be significantly more common than after other viral lower respiratory tract illnesses (Cohen et al., 2022), including influenza (Taquet et al., 2021). Therefore, it deserves more attention during research focused on many aspects of the SARS‐CoV‐2 infection (Llorente et al., 2022).

Discrepancies exist regarding the role of sex in the prevalence and the course of the post‐COVID fatigue (Janbazi et al., 2022; Sigfrid et al., 2021; Simani et al., 2021). Admittedly, some of the previous studies performed in different ethnic population showed that female sex was a risk factor for the development of persistent neuropsychiatric symptoms (Kim et al., 2022) and a long COVID syndrome, with nearly 70% of patients reporting fatigue (González‐Andrade, 2022; Mahmud et al., 2021). However, most data on sex‐related differences in the course of symptoms after the SARS‐CoV‐2 infection came from hospitalized patients (Cabrera Martimbianco et al., 2021; Munblit et al., 2021). For example, in a Danish cohort of 83 subjects previously hospitalized due to COVID‐19 and assessed using validated questionnaires 8 months after discharge, the risk of fatigue was associated with female sex in contrast to other factors, including the number of comorbidities (Schouborg et al., 2022). Surprisingly, outpatients compared to those hospitalized due to COVID‐19 had a higher fatigue rate not only in the acute phase of infection, but also within 30 days after discharge or diagnosis (Yoo et al., 2022). Analysis of even mild cases with COVID‐19 3 months after infection showed a high prevalence of fatigue, with 54.4% of women and 30.2% of men suffering from this syndrome (Maamar et al., 2022). In a national Saudi Arabian survey, with nearly 6000 respondents of whom 90% did not require hospitalization for COVID‐19, it was shown that female sex predicted a delayed return to the pre‐COVID health state (Tleyjeh et al., 2022). A Missouri cross‐sectional study showed that 43.3% of patients suffered from fatigue between 4 weeks and 6 months after the SARS‐CoV‐2 infection, with women experiencing significantly higher fatigue scores compared to men (Khatib et al., 2022). A detailed neuropsychological evaluation of 136 patients 8 months after COVID‐19 revealed that female sex was significantly associated only with the physical component of fatigue (Calabria et al., 2022). On the other hand, the evaluation of 226 COVID‐19 survivors from Belgium 3 and 12 months after hospital discharge showed that female sex was associated with the risk of persistent cognitive impairment but not fatigue (Lorent et al., 2022). No sex differences related to the post‐COVID fatigue were also found in recent studies assessing Swiss (Larsson et al., 2022) and Swedish populations (Diem et al., 2022). Moreover, to date, only few studies have presented details on the course of fatigue after COVID‐19, separately in women and men.

Therefore, we aimed to assess chronic fatigue symptoms and their timely course in non‐hospitalized patients with the SARS‐CoV‐2 infection, according to their sex.

## 2 MATERIALS AND METHODS

### 2.1 Fatigue assessment

**FIGURE 1:**
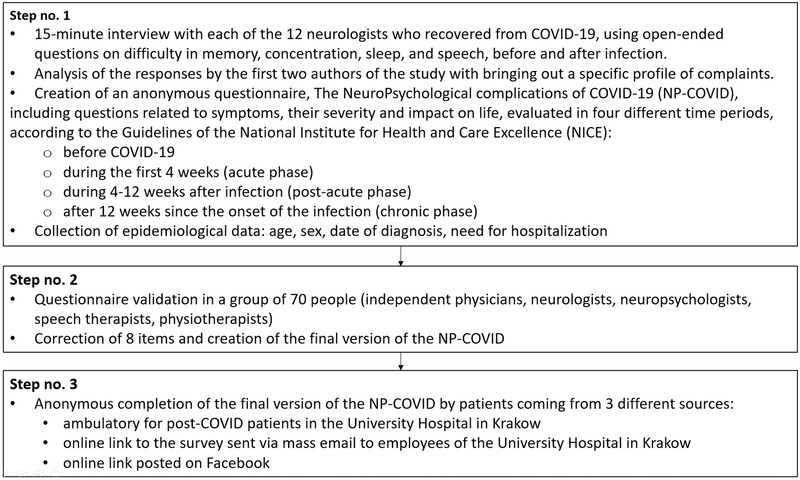
Flow chart showing the steps to create the questionnaire to assess the neuropsychological complications of COVID‐19

To prepare a short and precise clinical questionnaire that evaluated neuropsychological problems after the SARS‐CoV‐2 infection, we used an approach in three steps illustrated in Figure 1. In brief, based on the personal and professional experience of the interviewed neurologists who underwent COVID‐19, we created the questionnaire called “The NeuroPsychological complications of COVID‐19” (NP‐COVID), which was further validated in a group of 70 physicians and therapists (Figure 1). In the current study, we focused on fatigue that was retrospectively evaluated by patients in the second part of the NP‐COVID questionnaire. Patients were asked about eight symptoms (Table 1) that constituted the most commonly used and previously validated 1994 Centers for Disease Control and Prevention definition of the chronic fatigue syndrome (Fukuda et al., 1994; Lim & Son, 2020). Most of other definitions of the chronic fatigue syndrome also included the symptoms listed in Table 1 (Lim & Son, 2020). The presence of chronic fatigue symptoms was evaluated in four different time periods, according to the guidelines of the National Institute for Health and Care Excellence (NICE) (NICE et al., 2020; Shah et al., 2021): before COVID‐19, during the first 4 weeks of infection (acute phase), during 4–12 weeks after infection (post‐acute phase), and after 12 weeks since the infection onset (chronic phase; based also on the current literature regarding COVID‐19 [Nalbandian et al., 2021]) (Figure 1; NICE et al., 2020; Shah et al., 2021).

**TABLE 1:** Symptoms of chronic fatigue assessed by patients in the NeuroPsychological‐COVID questionnaire

Did you experience problems with:
1. Persistent fatigue, not caused by an effort, persisting after rest, decreasing life quality—if yes, did you also experience problems with:
2. Sore throat
3. Sensation of lymph node enlargement in the neck or armpit
4. Myalgia (muscle aches)
5. Arthralgia (inflammation of multiple joints)
6. Headache
7. Non‐restorative sleep
8. Prolonged postexercise fatigue

### 2.2 Patients

The following inclusion criteria were set for participation in the study: 18 years or older, more than 12 weeks since the onset of COVID‐19, infection diagnosis confirmed by viral RNA detection by the reverse transcription chain reaction (RT‐PCR) from a nasopharyngeal swab, and ability to write and read. Between April and August 2021, a total number of 660 anonymous NP‐COVID questionnaires were received from three sources: ambulatory for post‐COVID patients at the University Hospital in Krakow, online link to the survey sent via mass e‐mail to the employees of the University Hospital in Krakow, and online link posted on Facebook (Figure 1). After exclusion of questionnaires with incomplete data and previously hospitalized individuals due to the SARS‐CoV‐2 infection, 303 subjects were included in the final analysis.

### 2.3 Bioethics

We conducted this study in accordance with the Declaration of Helsinki and within the CRACoV‐HHS project (CRAcow in CoVid pandemics—Home, Hospital, and Staff), which received the approval of the Jagiellonian University Bioethics Committee, and integrated basic and clinical research in many medical disciplines, involving different populations of COVID‐19 evaluated through the personnel of the University Hospital in Krakow (Sydor et al., 2021). As the NP‐COVID questionnaires were filled out anonymously and after further consulting legal opinion, the collection of data in the current study did not require additional approval from the Bioethics Committee. Participants recruited in the ambulatory for the post‐COVID patients in the University Hospital in Krakow signed written informed consent before filling out the paper version of the NP‐COVID questionnaire. According to Polish law, no written consent is needed to obtain from patients who anonymously complete the online questionnaire; however, providing full information on the aim of such questionnaire is mandatory. Therefore, the collection of data through online anonymous link in the current study was performed in accordance with these legal guidelines. Additionally, since the research was of minimal risk to patient, the completion of the online questionnaire was evidence of consent to participate.

### 2.4 Statistics

Quantitative data were demonstrated as medians and interquartile ranges (IQRs) and compared using the Mann–Whitney *U*‐test, based on the non‐normal distribution found with the Shapiro–Wilk test. Qualitative data were described by numbers and percentages and compared using the *χ* ^2^ test or Fisher's exact test for independent variables and with the McNemar *χ* ^2^ test or Cochran's *Q* test for dependent variables. The association of sex with symptoms of chronic fatigue after COVID‐19 was expressed as odds ratio (OR) and 95% confidence interval (CI). For pairwise comparisons, the Bonferroni correction was used and the significance level was set below .008. A *p*‐value of <.05 was considered statistically significant for other comparisons. The internal consistency of the NP‐COVID questionnaire was evaluated using Cronbach's alpha resulting in a value of .931 that indicated acceptable internal consistency (Carden et al., 2018). STATISTICA version 13.0 (Statsoft Inc., Tulsa, OK) was used to perform the analyses.

## 3 RESULTS

The data that confirm the results of the current study can be obtained from the corresponding author upon reasonable request.

### 3.1 Study population

The study comprised 303 non‐hospitalized patients, including 79.53% women, and the median age was 36 years (IQR: 30–48 years), with no differences between men and women (35 [31–40] vs. 39 [30–49] years, respectively, *p* = .149). Patients were assessed retrospectively after a median of 30 (IQR: 23–35) weeks since the onset of symptoms.

### 3.2 The prevalence of multiple chronic fatigue symptoms after COVID‐19

Before COVID‐19, 31.46% of patients reported fatigue symptoms with 11.59%, 11.92%, 6.29%, and 1.66% demonstrating one to two, three to four, five to six, and seven to eight symptoms, respectively. Within 4 weeks after the onset of the disease, 88.45% of patients declared fatigue symptoms and the frequency of multiple positive responses increased to 8.91%, 26.07%, 42.90%, and 10.56% for one to two, three to four, five to six, and seven to eight symptoms, respectively (*p* = .018). After 12 weeks, 64.41% of the group had symptoms of chronic fatigue, while the prevalence of multiple symptoms remained high, 17.96%, 24.41%, 18.64%, and 3.39%, respectively (*p* = .023).

In women, a higher prevalence of one or more chronic fatigue symptoms was found in all time intervals after COVID‐19 compared to men (below 4 weeks *p* < .001; 4–12 weeks, *p* = .013; >12 weeks, *p* = .035). We did not observe sex differences in the number of chronic fatigue symptoms before the onset of COVID‐19 (*p* = .440) (Figure S1).

### 3.3 The burden of elements of chronic fatigue after COVID‐19

#### 3.3.1 Women

Within 4 weeks after COVID‐19, the prevalence of all symptoms increased (Table 2; Figure S2). During more than 12 weeks since the onset of symptoms, the frequency of persistent fatigue, caused or not by an effort, myalgia, headache, and non‐restorative sleep was partially reduced. The prevalence of sore throat, arthralgia, and self‐reported lymph node enlargement returned to the pre‐COVID‐19 levels.

**TABLE 2:** Symptoms of chronic fatigue during all time intervals assessed retrospectively

	Prior to COVID‐19			0–4 weeks			4–12 weeks			>12 weeks			*p*‐value vs. baseline (men; women)*		
	Men	Women	*p*‐value	Men	Women	*p*‐value	Men	Women	*p*‐value	Men	Women	*p*‐value	0–4 weeks	4–12 weeks	>12 weeks
1.1 Persistent fatigue, not caused by an effort, persisting after rest	13 (20.97)	59 (24.58)	0.551	48 (77.42)	214 (88.80)	0.020	41 (66.13)	191 (79.25)	0.030	29 (50.00)	148 (62.45)	0.083	<0.001; <0.001	<0.001; <0.001	<0.001; <0.001
1.2 Sore throat	2 (3.23)	12 (4.98)	0.742	14 (22.58)	88 (36.51)	0.039	5 (8.06)	47 (19.50)	0.037	3 (5.17)	21 (8.86)	0.434	0.001; <0.001	0.180; <0.001	0.563; 0.051
1.3 Self‐reported lymph node enlargement	1 (1.61)	8 (3.32)	0.691	5 (8.06)	43 (17.84)	0.078	3 (4.84)	32 (13.28)	0.075	0 (0.00)	14 (5.91)	0.080	0.102; <0.001	0.157; <0.001	0.317; 0.134
1.4 Myalgia	6 (9.68)	31 (12.86)	0.495	31 (50.00)	150 (62.24)	0.080	20 (32.26)	104 (43.15)	0.120	12 (20.69)	72 (30.38)	0.143	<0.001; <0.001	<0.001; <0.001	0.034; <0.001
1.5 Arthralgia	0 (0.00)	18 (7.47)	0.029	4 (6.45)	41 (17.01)	0.044	4 (6.45)	33 (13.69)	0.120	2 (3.45)	26 (10.97)	0.085	0.045; <0.001	0.046; 0.002	0.157; 0.102
1.6 Headache	9 (14.52)	60 (24.90)	0.082	32 (51.61)	177 (73.44)	<0.001	27 (43.55)	138 (57.26)	0.053	18 (31.03)	100 (42.19)	0.120	<0.001; <0.001	<0.001; <0.001	0.002; <0.001
1.7 Non‐restorative sleep	6 (9.68)	46 (19.09)	0.080	29 (46.77)	158 (65.56)	0.007	24 (38.71)	126 (52.28)	0.057	15 (25.86)	96 (40.51)	0.039	<0.001; <0.001	<0.001; <0.001	0.006; <0.001
1.8 Prolonged postexercise fatigue	6 (9.68)	42 (17.43)	0.137	43 (69.35)	195 (80.91)	0.048	39 (62.90)	160 (66.39)	0.607	23 (39.66)	119 (50.21)	0.149	<0.001; <0.001	<0.001; <0.001	<0.001; <0.001

*Note*: Data are presented as numbers (*n*) and percentages (%), *Cochran's *Q* test for dependent variable. The Bonferroni correction was used for pairwise comparisons, and the significance level was set below .008.

#### 3.3.2 Men

The prevalence of persistent effort‐related and unrelated fatigue, sore throat, myalgia, headache, and non‐restorative sleep increased within 4 weeks since the onset of COVID‐19 (questions 1, 2, 4, and 6–8, Table 2; Figure S2). We observed an increase in the number of patients with arthralgia and self‐reported lymph node enlargement, which remained statistically insignificant. Regarding the resolution of symptoms, the frequency of sore throat normalized in 4–12 weeks and myalgia in >12 weeks. Furthermore, persistent effort‐related and unrelated fatigue, headache, and non‐restorative sleep decreased during >12 weeks since the onset of infection, but without complete normalization.

### 3.4 Chronic fatigue symptoms in women and men

**FIGURE 2:**
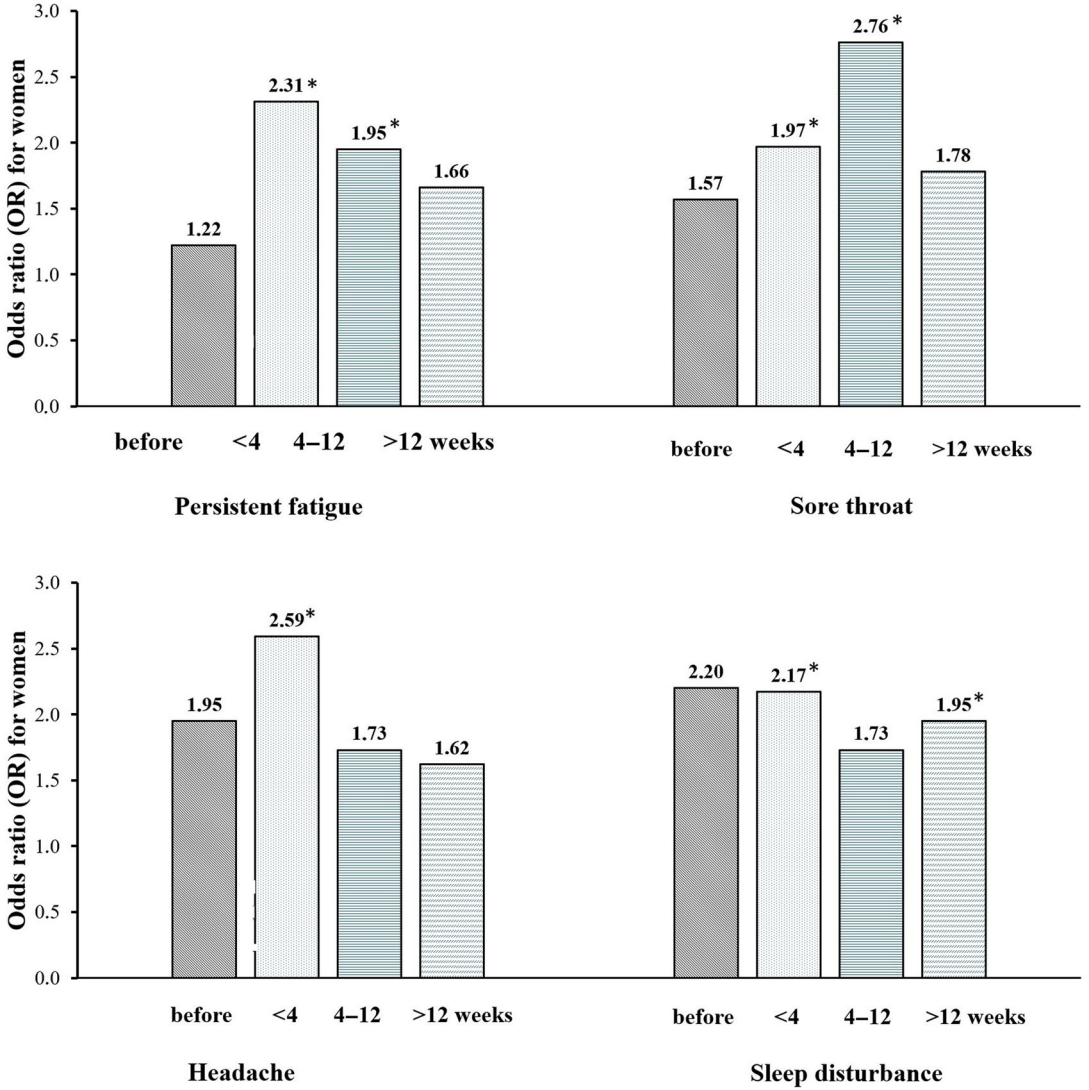
Risk of fatigue symptoms after COVID‐19 in women compared to men. OR, odds ratio

Persistent fatigue, not caused by an effort and persisting after rest, was more frequent in women throughout the 12‐week period since the onset of COVID‐19 compared to men (for <4 weeks, odds ratio [OR] = 2.31, 95% confidence interval [CI]: 1.13–4.73 and for 4–12 weeks, OR = 1.95, 95% CI: 1.06–3.61) (Figure 2). A higher prevalence of non‐restorative sleep was observed in women for more than 12 weeks (for 0–4 weeks, OR = 2.17, 95% CI: 1.23–3.81 and >12 weeks, OR = 1.95, 95% CI: 1.03–3.71). A difference in sore throat was observed to decrease in 12 weeks (OR = 1.97, 95% CI: 1.03–3.78 and OR = 2.76, 95% CI: 1.05–7.27 for <4 weeks and 4–12 weeks, respectively, for women compared to men). Sex differences in headache, arthralgia, and prolonged postexercise fatigue were temporarily observed only in the first 4 weeks (OR = 2.59, 95% CI: 1.45–4.60, OR = 2.97, 95% CI: 1.02–8.64, and OR = 1.87, 95% CI: 1.01–3.51, respectively). We did not observe differences between women and men in myalgia and self‐reported lymph node enlargement.

## 4 DISCUSSION

Our study is one of the first to demonstrate that the course of symptoms constituting COVID‐19‐associated chronic fatigue syndrome differs significantly between sexes. We were able to show that women more often suffered from persistent fatigue, not caused by an effort and not alleviated by rest, within 12 weeks since the onset of the SARS‐CoV‐2 infection. So far, it has been known that among patients with mild COVID‐19, fatigue (Miyazato et al., 2022) and malaise after exertion were correlated with female sex (Havdal et al., 2022). In relation to the post‐COVID fatigue, these sex differences were also found significant in studies with similar to ours cross‐sectional design (Fugazzaro et al., 2022), and in cohorts consisting of medical workers of whom most did not require hospitalization due to COVID‐19 (Sultana et al., 2021). Female sex was also found to predict the risk of post‐acute COVID‐19 sequelae, with fatigue being its most common symptom (Knight et al., 2022). Our study additionally revealed that the prevalence of non‐restorative sleep was higher in women after more than 3 months since the onset of infection. Indeed, previous studies showed that female sex was a risk factor for sleep disturbances after recovery from the acute phase of the SARS‐CoV‐2 infection, both in hospitalized and ambulatory cohorts (Pelà et al., 2022; Schou et al., 2021). We did not observe differences between sexes in relation to myalgia that resembled conclusions coming from 5‐month observation of Italian COVID‐19 survivors (Pelà et al., 2022). However, other researchers revealed that women followed 3 months after mild SARS‐CoV‐2 infection more often suffered not only from fatigue and sleep disturbances but also myalgia and headache (Kashif et al., 2021). In our study, we were unable to replicate findings related to headache; nevertheless, in the acute phase of infection this chronic fatigue symptom was more common in women, which stayed in line with previous research (Silverberg et al., 2022). Therefore, it seems that the course of symptoms constituting the chronic fatigue syndrome differs according to the sex of patient.

Our study confirmed that most patients during the acute phase of COVID‐19 experienced fatigue symptoms and after 12 weeks, besides improvement, the prevalence of multiple symptoms still remained high. We were also able to present details on the timely course of the post‐COVID fatigue. Most of the previous studies focused on the predictors of the post‐COVID fatigue and proved the role of female sex as a potential risk factor (Joli et al., 2022). Nevertheless, one retrospective study evaluating electronic health data from nearly 280,000 COVID‐19 survivors within 6 months after diagnosis showed that compared to the acute phase of infection, symptoms creating a long COVID syndrome more often coexisted with each other (Taquet et al., 2021). Regarding the chronic fatigue syndrome, we were able to show that in fact, multiple symptoms assessed by the NP‐COVID questionnaire co‐occurred with each other, even more than 12 weeks after the SARS‐CoV‐2 infection. Similarly, in a cohort of 2000 patients from five public hospitals in Spain, female sex was not only associated with fatigue 8 months after discharge, but also with at least three post‐COVID symptoms (Fernández‐de‐las‐Peñas et al., 2022). In a recent Brazilian study, the authors investigated the co‐occurrence of 30 symptoms of different body organs, and showed that fatigue, psychiatric, and cognitive manifestations were the most discriminatory for the so‐called post‐acute sequelae of the SARS‐CoV‐2 infection (Busatto et al., 2022). Similar to the results of our research, a previous large Spanish cohort study confirmed the tendency for fatigue to decrease over months since the infection onset (Fernández‐de‐las‐Peñas et al., 2022). On the other hand, a Korean prospective online survey showed that the prevalence of fatigue increased between 1 and 6 months after the acute phase of the SARS‐CoV‐2 infection and then gradually decreased; however, still around 15% of responders experienced this symptom as residual (Kim et al., 2022). This value was even higher in Egyptian COVID‐19 survivors (Kamal et al., 2021). In a study from four New York City hospitals, it was shown that between 6 and 12 months of follow‐up, there was an insignificant trend toward improving fatigue scores; however, the group consisted of patients with more severe COVID‐19, with a third of them previously needing intubation (Frontera et al., 2022). In a Norwegian questionnaire study, COVID‐19‐associated fatigue, which was more common in women, began to decrease after around 4 months (Stavem et al., 2021). On the contrary, in a recent meta‐analysis of 63 studies, comprising 250,000 patients with COVID‐19, it was shown that the prevalence of sleep disorders and fatigue did not decrease when comparing the observation time after COVID‐19 3–6 versus 6–9 months (Alkodaymi et al., 2022). Thus, a significant proportion of patients, besides initial improvement, still suffers from symptoms of the chronic fatigue syndrome even months after the onset of the SARS‐CoV‐2 infection, as was also confirmed in our study.

Our study has several important limitations. First, diagnostic criteria for chronic fatigue syndrome included more than 6 months of the duration of symptoms (Carod Artal, 2021). However, the definition of chronic post‐COVID fatigue for the purpose of this study remained consistent with the previous review of the literature (Nalbandian et al., 2021) and the NICE guidelines (Shah et al., 2021) that required the persistence of fatigue for more than 12 weeks. In fact, previous studies used different timelines to assess the post‐COVID symptoms, ranging from 2–3 months after the onset of infection (Raman et al., 2021) to even 12 months after recovery (Kim et al., 2022). Second, we used our own questionnaire created for the purpose of the study, tested in a small sample of physicians and therapists; however, it included the symptoms previously defining the chronic fatigue syndrome (Fukuda et al., 1994) and the most important elements of the post‐COVID fatigue, such as persistence and  reduction in the quality of life, that were proposed in other post‐COVID functional scales (Klok et al., 2020). Third, the results of the current study came only from symptoms retrospectively reported by patients that could have resulted in their underestimation, as questionnaires were completed only once 7 months after the onset of the disease. However, the prevalence of post‐COVID fatigue was comparable to previous studies (Lopez‐Leon et al., 2021). Fourth, we did not collect data related to possible other confounders, such as comorbidities.

To conclude, the course of post‐COVID fatigue differs significantly between non‐hospitalized women and men. Fatigue not caused by an effort and persisting after rest, non‐restorative sleep, and sore throat are observed more frequently in women compared to men. Besides some studies have shown the usefulness of mobile applications designed for cancer patients in treating fatigue due to COVID‐19 (Ruckser‐Scherb et al., 2022), future research is needed in order to better assess the course of post‐COVID fatigue and find appropriate treatment strategies (Manu, 2022).

## AUTHOR CONTRIBUTIONS

Iwona Mazurkiewicz conceptualized the idea of the study, wrote the original draft, curated the data, and designed the methodology. Zaneta Chatys‐Bogacka wrote the original draft, curated the data, and designed the methodology. Joanna Slowik designed the methodology and reviewed the draft. Alicja Klich‐Raczka and Malgorzata Fedyk‐Lukasik reviewed the draft. Agnieszka Slowik performed supervision, designed the methodology, and reviewed the draft. Marcin Wnuk conceptualized the idea of the study, performed supervision, and wrote and reviewed the draft. Leszek Drabik conceptualized the idea of the study, performed formal analysis, and wrote and reviewed the draft.

## CONFLICT OF INTEREST

The authors declare no conflict of interest.

### PEER REVIEW

The peer review history for this article is available at https://publons.com/publon/10.1002/brb3.2849.

## Supporting information

Supplemental Figure 1. Prevalence of fatigue symptoms after COVID‐19 in women and men within four time intervals assessed retrospectively
Supplemental Figure 2. Prevalence of fatigue symptoms after COVID‐19 in women and men

## Data Availability

The data that confirm the results of the current study can be obtained from the corresponding author upon reasonable request.

## References

[brb32849-bib-0001] Alkodaymi, M. S. , Omrani, O. A. , Fawzy, N. A. , Shaar, B. A. , Almamlouk, R. , Riaz, M. , Obeidat, M. , Obeidat, Y. , Gerberi, D. , Taha, R. M. , Kashour, Z. , Kashour, T. , Berbari, E. F. , Alkattan, K. , & Tleyjeh, I. M. (2022). Prevalence of post‐acute COVID‐19 syndrome symptoms at different follow‐up periods: A systematic review and meta‐analysis. Clinical Microbiology and Infection, 28, 657–666. 35124265 10.1016/j.cmi.2022.01.014 PMC8812092

[brb32849-bib-0002] Bliddal, S. , Banasik, K. , Pedersen, O. B. , Nissen, J. , Cantwell, L. , Schwinn, M. , Tulstrup, M. , Westergaard, D. , Ullum, H. , Brunak, S. , Tommerup, N. , Feenstra, B. , Geller, F. , Ostrowski, S. R. , Grønbæk, K. , Nielsen, C. H. , Nielsen, S. D. , & Feldt‐Rasmussen, U. (2021). Acute and persistent symptoms in non‐hospitalized PCR‐confirmed COVID‐19 patients. Scientific Reports, 11, 13153. 34162913 10.1038/s41598-021-92045-x PMC8222239

[brb32849-bib-0003] Busatto, G. F. , de Araujo, A. L. , Castaldelli‐Maia, J. M. , Damiano, R. F. , Imamura, M. , Guedes, B. F. , de Rezende Pinna, F. , Sawamura, M. V. Y. , Mancini, M. C. , da Silva, K. R. , Garcia, M. L. , Sumita, N. , Brunoni, A. R. , da Silva Duarte, A. J. , Burdmann, E. A. , Kallas, E. G. , Cerri, G. G. , Nitrini, R. , Bento, R. F. , … HCFMUSP Covid‐19 Study Group . (2022). Post‐acute sequelae of SARS‐CoV‐2 infection: Relationship of central nervous system manifestations with physical disability and systemic inflammation. Psychological Medicine, 52(12), 2387–2398. 35521752 10.1017/S0033291722001374 PMC9151630

[brb32849-bib-0004] Cabrera Martimbianco, A. L. , Pacheco, R. L. , Bagattini, Â. M. , & Riera, R. (2021). Frequency, signs and symptoms, and criteria adopted for long COVID‐19: A systematic review. International Journal of Clinical Practice, 75, 1–16. 10.1111/ijcp.14357 PMC8236920 33977626

[brb32849-bib-0005] Calabria, M. , García, C. , Nicholas, S. , Pons, C. , Antonio, J. , Anson, B. G. , Estévez García, M. , Belvís, R. , Morollón, N. , Vera Igual, J. , Mur, I. , Pomar, V. , & Domingo, P. (2022). Post‐COVID‐19 fatigue : The contribution of cognitive and neuropsychiatric symptoms. Journal of Neurology, 269(8), 3990–3999. 35488918 10.1007/s00415-022-11141-8 PMC9055007

[brb32849-bib-0006] Carden, S. , Camper, T. , & Holtzman, N. (2018). Cronbach's alpha under insufficient effort responding: An analytic approach. Stats, 2, 1–14.

[brb32849-bib-0007] Carod Artal, F. J. (2021). Síndrome post‐COVID‐19: Epidemiología, criterios diagnósticos y mecanismos patogénicos implicados. Revista De Neurologia, 72, 384–396. 34042167 10.33588/rn.7211.2021230

[brb32849-bib-0008] Chen, C. , Haupert, S. R. , Zimmermann, L. , Shi, X. , Fritsche, L. G. , & Mukherjee, B. (2022). Global prevalence of post COVID‐19 condition or long COVID: A meta‐analysis and systematic review. Journal of Infectious Diseases, 226(9), 1593–1607. 35429399 10.1093/infdis/jiac136 PMC9047189

[brb32849-bib-0009] Cohen, K. , Ren, S. , Heath, K. , Dasmariñas, M. C. , Jubilo, K. G. , Guo, Y. , Lipsitch, M. , & Daugherty, S. E. (2022). Risk of persistent and new clinical sequelae among adults aged 65 years and older during the post‐acute phase of SARS‐CoV‐2 infection: Retrospective cohort study. BMJ, 376, 1–12. 10.1136/bmj-2021-068414 PMC8828141 35140117

[brb32849-bib-0010] Diem, L. , Fregolente‐Gomes, L. , Warncke, J. D. , Hammer, H. , Friedli, C. , Kamber, N. , Jung, S. , Bigi, S. , Funke‐Chambour, M. , Chan, A. , Bassetti, C. L. , Salmen, A. , & Hoepner, R. (2022). Fatigue in post‐covid‐19 syndrome: Clinical phenomenology, comorbidities and association with initial course of COVID‐19. Journal of Central Nervous System Disease, 14, 117957352211027. 10.1177/11795735221102727 PMC9130865 35633835

[brb32849-bib-0011] Fernández‐de‐las‐Peñas, C. , Martín‐Guerrero, J. D. , Cancela‐Cilleruelo, I. , Moro‐López‐Menchero, P. , & Pellicer‐Valero, O. J. (2022). Exploring the recovery curve for long‐term post‐COVID dyspnea and fatigue. European Journal of Internal Medicine, 101, 120–123. 35490087 10.1016/j.ejim.2022.03.036 PMC9046058

[brb32849-bib-0012] Fernández‐de‐las‐Peñas, C. , Martín‐Guerrero, J. D. , Pellicer‐Valero, Ó. J. , Navarro‐Pardo, E. , Gómez‐Mayordomo, V. , Cuadrado, M. L. , Arias‐Navalón, J. A. , Cigarán‐Méndez, M. , Hernández‐Barrera, V. , & Arendt‐Nielsen, L. (2022). Female sex is a risk factor associated with long‐term post‐COVID related‐symptoms but not with COVID‐19 symptoms: The LONG‐COVID‐EXP‐CM multicenter study. Journal of Clinical Medicine, 11, 413. 35054108 10.3390/jcm11020413 PMC8778106

[brb32849-bib-0013] Fernández‐de‐las‐Peñas, C. , Palacios‐Ceña, D. , Gómez‐Mayordomo, V. , Florencio, L. L. , Cuadrado, M. L. , Plaza‐Manzano, G. , & Navarro‐Santana, M. (2021). Prevalence of post‐COVID‐19 symptoms in hospitalized and non‐hospitalized COVID‐19 survivors: A systematic review and meta‐analysis. European Journal of Internal Medicine, 92, 55–70. 34167876 10.1016/j.ejim.2021.06.009 PMC8206636

[brb32849-bib-0014] Fernández‐Lázaro, D. , Sánchez‐Serrano, N. , Mielgo‐Ayuso, J. , García‐Hernández, J. L. , González‐Bernal, J. J. , & Seco‐Calvo, J. (2021). Long COVID a new derivative in the chaos of SARS‐CoV‐2 infection: The emergent pandemic? Journal of Clinical Medicine, 10, 1–18. 10.3390/jcm10245799 PMC8708091 34945095

[brb32849-bib-0015] Frontera, J. A. , Yang, D. , Medicherla, C. , Baskharoun, S. , Bauman, K. , Bell, L. , Bhagat, D. , Bondi, S. , Chervinsky, A. , Dygert, L. , Fuchs, B. , Gratch, D. , Hasanaj, L. , Horng, J. , Huang, J. , Jauregui, R. , Ji, Y. , Kahn, D. E. , Koch, E. , … Galetta, S. (2022). Trajectories of neurologic recovery 12 months after hospitalization for COVID‐19: A prospective longitudinal study. Neurology, 99(1), e33–e45. 35314503 10.1212/WNL.0000000000200356 PMC9259089

[brb32849-bib-0016] Fugazzaro, S. , Denti, M. , Mainini, C. , Accogli, M. A. , Bedogni, G. , Ghizzoni, D. , Bertolini, A. , Esseroukh, O. , Gualdi, C. , Schiavi, M. , Braglia, L. , & Costi, S. (2022). Sex differences and rehabilitation needs after hospital discharge for COVID‐19: An Italian cross‐sectional study. BMJ Open, 12, e055308. 10.1136/bmjopen-2021-055308 PMC9118361 35584875

[brb32849-bib-0017] Fukuda, K. , Straus, S. , Hickie, I. , Sharpe, M. , Dobbins, J. , & Komaroff, A. (1994). The chronic fatigue syndrome: A comprehensive approach to its definition and study. International Chronic Fatigue Syndrome Study Group. Annals of Internal Medicine, 121, 953–959. 7978722 10.7326/0003-4819-121-12-199412150-00009

[brb32849-bib-0018] González‐Andrade, F. (2022). Post‐COVID‐19 conditions in Ecuadorian patients: An observational study. The Lancet Regional Health ‐ Americas, 5, 100088. 34870261 10.1016/j.lana.2021.100088 PMC8633922

[brb32849-bib-0019] Havdal, L. B. , Berven, L. L. , Selvakumar, J. , Stiansen‐Sonerud, T. , Leegaard, T. M. , Tjade, T. , Zetterberg, H. , Blennow, K. , & Wyller, V. B. B. (2022). Neurological involvement in COVID‐19 among non‐hospitalized adolescents and young adults. Frontiers in Neurology, 13, 915712. 35812102 10.3389/fneur.2022.915712 PMC9257204

[brb32849-bib-0020] Healey, Q. , Sheikh, A. , Daines, L. , & Vasileiou, E. (2022). Symptoms and signs of long COVID: A rapid review and meta‐analysis. Journal of Global Health, 12, 05014. 35596571 10.7189/jogh.12.05014 PMC9125197

[brb32849-bib-0021] Hellwig, S. , & Domschke, K. (2022). Post‐COVID Syndrome – Fokus fatigue. Der Nervenarzt, 93(8), 788–796. 35606656 10.1007/s00115-022-01306-1 PMC9126432

[brb32849-bib-0022] Janbazi, L. , Kazemian, A. , Mansouri, K. , Madani, S. P. , Yousefi, N. , Vahedifard, F. , & Raissi, G. (2022). The incidence and characteristics of chronic pain and fatigue after 12 months later admitting with COVID‐19; the post‐ COVID 19 syndrome. American Journal of Physical Medicine & Rehabilitation, 10.1097/PHM.0000000000002030 35473921

[brb32849-bib-0023] Joli, J. , Buck, P. , Zipfel, S. , & Stengel, A. (2022). Post‐COVID‐19 fatigue: A systematic review. Frontiers in Psychiatry, 13, 947973. 36032234 10.3389/fpsyt.2022.947973 PMC9403611

[brb32849-bib-0024] Kamal, M. , Abo Omirah, M. , Hussein, A. , & Saeed, H. (2021). Assessment and characterization of post‐COVID‐19 manifestations. International Journal of Clinical Practice, 75, e13746. 32991035 10.1111/ijcp.13746 PMC7536922

[brb32849-bib-0025] Kashif, A. , Chaudhry, M. , Fayyaz, T. , Abdullah, M. , Malik, A. , Anwer, J. M. A. , Inam, S. H. A. , Fatima, T. , Iqbal, N. , & Shoaib, K. (2021). Follow‐up of COVID‐19 recovered patients with mild disease. Scientific Reports, 11, 1–5. 34183705 10.1038/s41598-021-92717-8 PMC8239036

[brb32849-bib-0026] Khatib, S. , Sabobeh, T. , Habib, A. , John, S. , Gomez, R. , Sivasankar, S. , & Masoud, A. (2022). Post‐COVID‐19 fatigue as a major health problem: A cross‐sectional study from Missouri, USA. Irish Journal of Medical Science, 1–7. 10.1007/s11845-022-03011-z PMC9013544 35434772

[brb32849-bib-0027] Kim, Y. , Bitna‐Ha , Kim, S. W. , Chang, H. H. , Kwon, K. T. , Bae, S. , & Hwang, S. (2022). Post‐acute COVID‐19 syndrome in patients after 12 months from COVID‐19 infection in Korea. BMC Infectious Diseases, 22, 1–12. 35086489 10.1186/s12879-022-07062-6 PMC8793328

[brb32849-bib-0028] Klok, F. A. , Boon, G. , Barco, S. , Endres, M. , Miranda Geelhoed, J. J. , Knauss, S. , Rezek, S. A. , Spruit, M. A. , Vehreschild, J. , & Siegerink, B. (2020). The post‐COVID‐19 functional status scale: A tool to measure functional status over time after COVID‐19. European Respiratory Journal, 56, 10–12. 10.1183/13993003.01494-2020 PMC7236834 32398306

[brb32849-bib-0029] Knight, D. R. T. , Munipalli, B. , Logvinov, I. I. , Halkar, M. G. , Mitri, G. , Dabrh, A. M. A. , & Hines, S. L. (2022). Perception, prevalence, and prediction of severe infection and post‐acute sequelae of COVID‐19. American Journal of the Medical Sciences, 363, 295–304. 35016849 10.1016/j.amjms.2022.01.002 PMC8743283

[brb32849-bib-0030] Larsson, A. C. , Engwall, M. , Palstam, A. , & Persson, H. C. (2022). Self‐assessed aspects of health 3 months after COVID‐19 hospitalization—A Swedish cross‐sectional study. International Journal of Environmental Research and Public Health, 19, 8020. 35805677 10.3390/ijerph19138020 PMC9265939

[brb32849-bib-0031] Lim, E. J. , & Son, C. G. (2020). Review of case definitions for myalgic encephalomyelitis/chronic fatigue syndrome (ME/CFS). Journal of Translational Medicine, 18, 289. 32727489 10.1186/s12967-020-02455-0 PMC7391812

[brb32849-bib-0032] Llorente, B. C. , López, A. M. C. , Sánchez, R. H. , & Hernández Gutierrez, C. (2022). Protocolo diagnóstico de las manifestaciones crónicas de la COVID‐19. Medicine, 13, 3256–3260. 35582695 10.1016/j.med.2022.05.007 PMC9097968

[brb32849-bib-0033] Logue, J. K. , Franko, N. M. , McCulloch, D. J. , McDonald, D. , Magedson, A. , Wolf, C. R. , & Chu, H. Y. (2021). Sequelae in adults at 6 months after COVID‐19 infection. JAMA Network Open, 4, e210830. 33606031 10.1001/jamanetworkopen.2021.0830 PMC7896197

[brb32849-bib-0034] Lopez‐Leon, S. , Wegman‐Ostrosky, T. , Perelman, C. , Sepulveda, R. , Rebolledo, P. A. , Cuapio, A. , & Villapol, S. (2021). More than 50 long‐term effects of COVID‐19: A systematic review and meta‐analysis. Scientific Reports, 11, 1–22. 34373540 10.1038/s41598-021-95565-8 PMC8352980

[brb32849-bib-0035] Lorent, N. , Vande Weygaerde, Y. , Claeys, E. , Guler Caamano Fajardo, I. , De Vos, N. , De Wever, W. , Salhi, B. , Gyselinck, I. , Bosteels, C. , Lambrecht, B. N. , Everaerts, S. , Verschraegen, S. , Schepers, C. , Demeyer, H. , Heyns, A. , Depuydt, P. , Oeyen, S. , Van Bleyenbergh, P. , Godinas, L. , … Van Braeckel, E. (2022). Prospective longitudinal evaluation of hospitalised COVID‐19 survivors 3 and 12 months after discharge. ERJ Open Research, 8, 00004–02022. 35415186 10.1183/23120541.00004-2022 PMC8994962

[brb32849-bib-0036] Maamar, M. , Artime, A. , Pariente, E. , Fierro, P. , Ruiz, Y. , Gutiérrez, S. , Tobalina, M. , Díaz‐Salazar, S. , Ramos, C. , Olmos, J. M. , & Hernández, J. L. (2022). Post‐COVID‐19 syndrome, low‐grade inflammation and inflammatory markers: A cross‐sectional study. Current Medical Research and Opinion, 38(6), 901–909. 35166141 10.1080/03007995.2022.2042991 PMC8935459

[brb32849-bib-0037] Mahmud, R. , Rahman, M. M. , Rassel, M. A. , Monayem, F. B. , Sayeed, S. , Islam, M. S. , & Islam, M. M. (2021). Post‐COVID‐19 syndrome among symptomatic COVID‐19 patients: A prospective cohort study in a tertiary care center of Bangladesh. PLoS ONE, 16, 1–13. 10.1371/journal.pone.0249644 PMC8031743 33831043

[brb32849-bib-0038] Manu, P. (2022). Repurposing drugs for post‐COVID‐19 fatigue syndrome: Methylphenidate, duloxetine, and brexpiprazole. American Journal of Therapeutics, 29, e229–e230. 35389574 10.1097/MJT.0000000000001471

[brb32849-bib-0039] Miyazato, Y. , Tsuzuki, S. , Morioka, S. , Terada, M. , Kutsuna, S. , Saito, S. , Shimanishi, Y. , Takahashi, K. , Sanada, M. , Akashi, M. , Kuge, C. , Osanai, Y. , Tanaka, K. , Suzuki, M. , Hayakawa, K. , & Ohmagari, N. (2022). Factors associated with development and persistence of post‐COVID conditions: A cross‐sectional study. Journal of Infection and Chemotherapy, 28(9), 1242–1248. 35595598 10.1016/j.jiac.2022.04.025 PMC9114006

[brb32849-bib-0040] Munblit, D. , Bobkova, P. , Spiridonova, E. , Shikhaleva, A. , Gamirova, A. , Blyuss, O. , Nekliudov, N. , Bugaeva, P. , Andreeva, M. , DunnGalvin, A. , Comberiati, P. , Apfelbacher, C. , Genuneit, J. , Avdeev, S. , Kapustina, V. , Guekht, A. , Fomin, V. , Svistunov, A. A. , Timashev, P. , … Sechenov StopCOVID Research Team . (2021). Incidence and risk factors for persistent symptoms in adults previously hospitalized for COVID‐19. Clinical and Experimental Allergy, 51, 1107–1120. 34351016 10.1111/cea.13997 PMC8444748

[brb32849-bib-0041] Nalbandian, A. , Sehgal, K. , Gupta, A. , Madhavan, M. V. , McGroder, C. , Stevens, J. S. , Cook, J. R. , Nordvig, A. S. , Shalev, D. , Sehrawat, T. S. , Ahluwalia, N. , Bikdeli, B. , Dietz, D. , Der‐Nigoghossian, C. , Liyanage‐Don, N. , Rosner, G. F. , Bernstein, E. J. , Mohan, S. , Beckley, A. A. , … Wan, E. Y. (2021). Post‐acute COVID‐19 syndrome. Nature Medicine, 27, 601–615. 10.1038/s41591-021-01283-z PMC8893149 33753937

[brb32849-bib-0042] National Institute for Health and Care Excellence (NICE) , Royal College of General Practitioners (RCGP) , & Scottish Intercollegiate Guidelines Network (SIGN) . (2020). COVID‐19 rapid guideline: Managing the long‐term effects of COVID‐19 . Author. 33555768

[brb32849-bib-0043] Pelà, G. , Goldoni, M. , Solinas, E. , Cavalli, C. , Tagliaferri, S. , Ranzieri, S. , Frizzelli, A. , Marchi, L. , Mori, P. A. , Majori, M. , Aiello, M. , Corradi, M. , & Chetta, A. (2022). Sex‐related differences in long‐COVID‐19 syndrome. Journal of Women's Health, 31, 620–630. 10.1089/jwh.2021.0411 35333613

[brb32849-bib-0044] Petersen, M. S. , Kristiansen, M. F. , Hanusson, K. D. , Danielsen, M. E. , á Steig, B. , Gaini, S. , Strøm, M. , & Weihe, P. (2021). Long COVID in the Faroe Islands: A longitudinal study among nonhospitalized patients. Clinical Infectious Diseases, 73, e4058–e4063. 33252665 10.1093/cid/ciaa1792 PMC7799340

[brb32849-bib-0045] Raman, B. , Cassar, M. P. , Tunnicliffe, E. M. , Filippini, N. , Griffanti, L. , Alfaro‐Almagro, F. , Okell, T. , Sheerin, F. , Xie, C. , Mahmod, M. , Mózes, F. E. , Lewandowski, A. J. , Ohuma, E. O. , Holdsworth, D. , Lamlum, H. , Woodman, M. J. , Krasopoulos, C. , Mills, R. , McConnell, F. A. K. , … Neubauer, S. (2021). Medium‐term effects of SARS‐CoV‐2 infection on multiple vital organs, exercise capacity, cognition, quality of life and mental health, post‐hospital discharge. EClinicalMedicine, 31, 100683. 33490928 10.1016/j.eclinm.2020.100683 PMC7808914

[brb32849-bib-0046] Ruckser‐Scherb, R. , Gassner, J. , & Himmelbauer, C. (2022). Fostering fatigue‐management in people with post‐acute COVID‐19 syndrome ‐ Experiences with the “Untire” app. Studies in Health Technology and Informatics, 293, 47–51. 35592959 10.3233/SHTI220346

[brb32849-bib-0047] Schou, T. M. , Joca, S. , Wegener, G. , & Bay‐Richter, C. (2021). Psychiatric and neuropsychiatric sequelae of COVID‐19 – A systematic review. Brain, Behavior, and Immunity, 97, 328–348. 34339806 10.1016/j.bbi.2021.07.018 PMC8363196

[brb32849-bib-0048] Schouborg, L. B. , Molsted, S. , Lendorf, M. E. , Hegelund, M. H. , Ryrsø, C. K. , Sommer, D. H. , Kolte, L. , Nolsöe, R. L. , Pedersen, T. I. , Harboe, Z. B. , Browatzki, A. , Brandi, L. , Krog, S. M. , Bestle, M. H. , Jørgensen, I. M. , Jensen, T. Ø. , Fischer, T. K. , Pedersen‐Bjergaard, U. , & Lindegaard, B. (2022). Risk factors for fatigue and impaired function eight months after hospital admission with COVID‐19. Danish Medical Journal, 69, 1–10. 35319451

[brb32849-bib-0049] Shah, W. , Hillman, T. , Playford, E. D. , & Hishmeh, L. (2021). Managing the long term effects of covid‐19: Summary of NICE, SIGN, and RCGP rapid guideline. BMJ, 372, 10–13. 10.1136/bmj.n136 33483331

[brb32849-bib-0050] Sigfrid, L. , Drake, T. M. , Pauley, E. , Jesudason, E. C. , Olliaro, P. , Lim, W. S. , Gillesen, A. , Berry, C. , Lowe, D. J. , McPeake, J. , Lone, N. , Munblit, D. , Cevik, M. , Casey, A. , Bannister, P. , Russell, C. D. , Goodwin, L. , Ho, A. , Turtle, L. , … ISARIC4C Investigators . (2021). Long Covid in adults discharged from UK hospitals after Covid‐19: A prospective, multicentre cohort study using the ISARIC WHO Clinical Characterisation Protocol. The Lancet Regional Health ‐ Europe, 8, 100186. 34386785 10.1016/j.lanepe.2021.100186 PMC8343377

[brb32849-bib-0051] Silverberg, J. I. , Zyskind, I. , Naiditch, H. , Zimmerman, J. , Glatt, A. E. , Pinter, A. , Theel, E. S. , Joyner, M. J. , Hill, D. A. , Lieberman, M. R. , Bigajer, E. , Stok, D. , Frank, E. , & Rosenberg, A. Z. (2022). Predictors of chronic COVID‐19 symptoms in a community‐based cohort of adults. PLoS ONE, 17, e0271310. 35925904 10.1371/journal.pone.0271310 PMC9352033

[brb32849-bib-0052] Simani, L. , Ramezani, M. , Darazam, I. A. , Sagharichi, M. , Aalipour, M. A. , Ghorbani, F. , & Pakdaman, H. (2021). Prevalence and correlates of chronic fatigue syndrome and post‐traumatic stress disorder after the outbreak of the COVID‐19. Journal of Neurovirology, 27, 154–159. 33528827 10.1007/s13365-021-00949-1 PMC7852482

[brb32849-bib-0053] Stavem, K. , Ghanima, W. , Olsen, M. K. , Gilboe, H. M. , & Einvik, G. (2021). Prevalence and determinants of fatigue after covid‐19 in non‐hospitalized subjects: A population‐based study. International Journal of Environmental Research and Public Health, 18, 1–11. 10.3390/ijerph18042030 PMC7921928 33669714

[brb32849-bib-0054] Sultana, S. , Islam, M. T. , Salwa, M. , Zakir Hossain, S. M. , Hasan, M. N. , Masum, A. A. , Khan, A. H. , Khan, M. M. H. , & Haque, M. A. (2021). Duration and risk factors of post‐COVID symptoms following recovery among the medical doctors in Bangladesh. Cureus, 13, 1–8. 10.7759/cureus.15351 PMC8245646 34239785

[brb32849-bib-0055] Sydor, W. , Wizner, B. , Strach, M. , Bociąga‐Jasik, M. , Mydel, K. , Olszanecka, A. , Sanak, M. , Małecki, M. , Wójkowska‐Mach, J. , Chrzan, R. , Garlicki, A. , Gosiewski, T. , Krzanowski, M. , Surowiec, J. , Bednarz, S. , Jędrychowski, M. , & Grodzicki, T. (2021). CRACoV‐HHS: An interdisciplinary project for multi‐specialist hospital and non‐hospital care for patients with SARS‐CoV‐2 infection as well hospital staff assessment for infection exposure. Folia Medica Cracoviensia, 61, 5–44. 10.24425/fmc.2021.140002 35180200

[brb32849-bib-0056] Taquet, M. , Dercon, Q. , Luciano, S. , Geddes, J. R. , Husain, M. , & Harrison, P. J. (2021). Incidence, co‐occurrence, and evolution of long‐COVID features: A 6‐month retrospective cohort study of 273,618 survivors of COVID‐19. PLoS Medicine, 18, 1–22. 10.1371/journal.pmed.1003773 PMC8478214 34582441

[brb32849-bib-0057] Tleyjeh, I. M. , Kashour, T. , Riaz, M. , Amer, S. A. , AlSwaidan, N. , Almutairi, L. , Halwani, R. , & Assiri, A. (2022). Persistent COVID‐19 symptoms at least one month after diagnosis: A national survey. Journal of Infection and Public Health, 15, 578–585. 35477145 10.1016/j.jiph.2022.04.006 PMC9020835

[brb32849-bib-0058] Yoo, S. M. , Liu, T. C. , Motwani, Y. , Sim, M. S. , Viswanathan, N. , Samras, N. , Hsu, F. , & Wenger, N. S. (2022). Factors associated with post‐acute sequelae of SARS‐CoV‐2 (PASC) after diagnosis of symptomatic COVID‐19 in the inpatient and outpatient setting in a diverse cohort. Journal of General Internal Medicine, 37, 1988–1995. 35391623 10.1007/s11606-022-07523-3 PMC8989256

[brb32849-bib-0059] Zhu, N. , Zhang, D. , Wang, W. , Li, X. , Yang, B. , Song, J. , Zhao, X. , Huang, B. , Shi, W. , Lu, R. , Niu, P. , Zhan, F. , Ma, X. , Wang, D. , Xu, W. , Wu, G. , Gao, G. F. , Tan, W. , & China Novel Coronavirus Investigating and Research Team . (2020). A novel coronavirus from patients with pneumonia in China, 2019. New England Journal of Medicine, 382, 727–733. 31978945 10.1056/NEJMoa2001017 PMC7092803

