# Supplementary material for: Fatigue after COVID‐19 in non‐hospitalized patients according to sex
Source: Brain Behav. 2023 Jan 9;13(2):e2849. doi: 10.1002/brb3.2849 (PMC9927850; doi:10.1002/brb3.2849)

**Supplemental Figure 1.** Prevalence of fatigue symptoms after COVID-19 in women and men within four time intervals assessed retrospectively. Colors correspond to a percentage of patients with multiple positive responses to eight questions of the questionnaire.


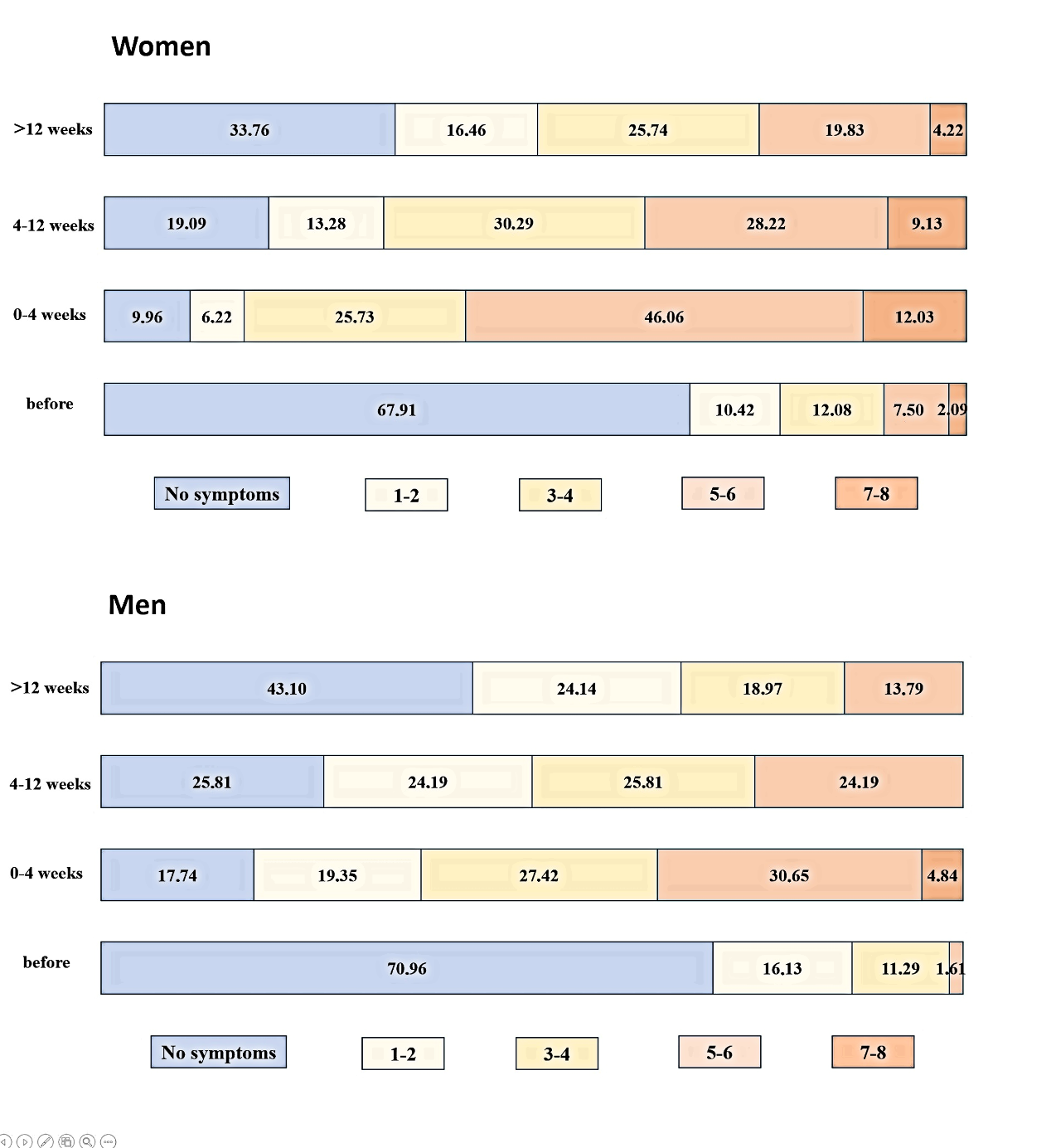


**Supplemental Figure 2**. Prevalence of fatigue symptoms after COVID-19 in women and men. The colors correspond to a percentage of patients with positive responses to questions 1-8. The P-values (<0.008) are Bonferroni corrected for multiple comparisons, * vs baseline, #vs < 4 weeks, $vs 4-12 weeks.


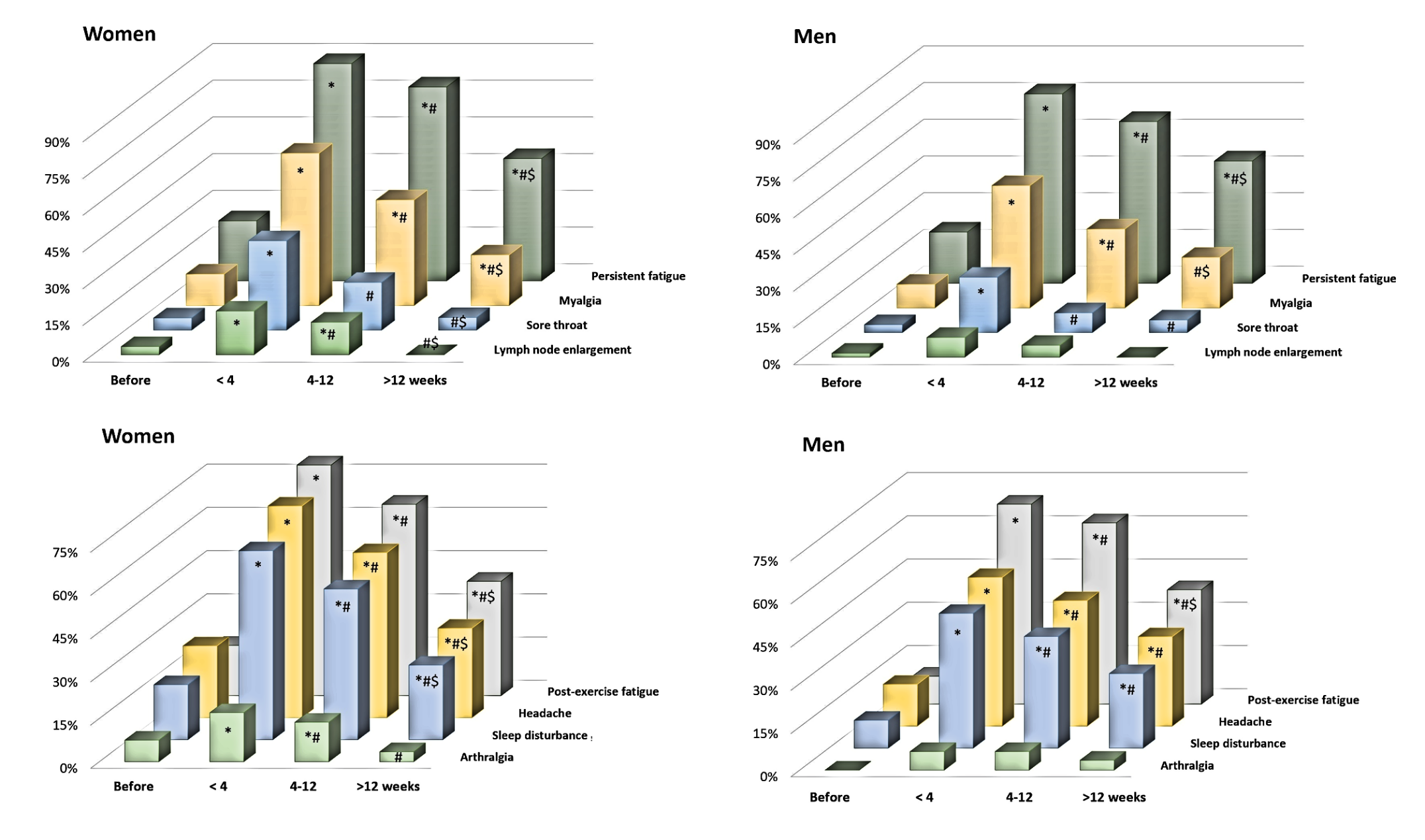

Supplement: Supplementary file 1 — Supplemental Figure 1. Prevalence of fatigue symptoms after COVID‐19 in women and men within four time intervals assessed retrospectively Supplemental Figure 2. Prevalence of fatigue symptoms after COVID‐19 in women and men [file BRB3-13-e2849-s001.docx]
